# Effect of citral on mouse hepatic cytochrome P450 enzymes

**DOI:** 10.1080/13880209.2018.1470191

**Published:** 2018-07-03

**Authors:** Huaqiao Tang, Nana Long, Min Dai, Lin Lin, Jianlong Li, Fenghui Sun, Lijuan Guo

**Affiliations:** a School of Laboratory Medicine, Chengdu Medical College, Chengdu, Sichuan, PR China;; b Laboratory of Veterinary Drug Residue Prevention and Control Technology of Animal-derived Food, Chengdu Medical College, Chengdu, Sichuan, PR China

**Keywords:** Drug–drug interaction, drug metabolism, liver damage, oxidative stress

## Abstract

**Context:** Citral is used as a potential natural treatment for various infectious diseases.

**Objective:** To examine the effect of citral on the mRNA expression and activities of cytochrome P450 (CYP450) enzymes and establish the relationship between citral-induced liver injury and oxidative stress.

**Materials and methods:** ICR mice were randomly divided into citral (20, 200, and 2000 mg/kglow), Tween-80, and control groups (0.9% saline), 10 mice in each group. The citral-treated groups were intragastrically administered citral for 3 d, control groups treated with 0.5% Tween-80 and 0.9% saline in the same way. Liver injury and CYP450 enzymes were analyzed by analyzing the histopathological changes and the changes of related enzymes.

**Results:** Citral treatment (2000 mg/kg) for 3 d increased serum glutamic pyruvic transaminase and glutamic oxaloacetic transaminase levels, as well as glutathione, gydroxyl radicals, malonaldehyde and total superoxide dismutase contents, but decreased the content of total antioxidant capacity. In doses of 20 and 200 mg/kg groups mice, the contents of NO were decreased significantly and other changes were similar to the 2000 mg/kg group mice, but the liver damage was most severe in the 2000 mg/kg group. Citral induced the mRNA expression and activities of CYP450 1A2, 2D22, and 2E1 in the liver of mice at doses of 20 and 200 mg/kg. There were no changes in testing indexes in Tween-80 treated group mice. Due to its toxic effects, the CYP induction effect of citral negatively correlated with its dose. Although the mRNA expression of CYP450 3A11 was induced by citral, its activity was not affected by low and moderate doses of citral. CYP450 3A11 activity was significantly decreased by high-dose citral.

**Conclusions:** Citral is hepatotoxic and induced oxidative stress in higher dose, which has a negative effect on CYP450 enzymes. These data suggest caution needs to be taken in order to avoid citral-drug interactions in human beings.

## Introduction

Plant-based natural products are important sources of drugs and food supplements. The antibacterial, antiviral, antitumor, and antioxidant activities of several plant components have been reported (Narender 2012; Medina-Franco 2013; Luo et al. 2014). Natural products isolated from plants have contributed significantly to the treatment of serious human diseases and have been used as drugs for thousands of years in China.

Citral (3,7-dimethyl-2,6-octadienal) is one of the most important open-chain monoterpenoids. It is present in volatile oils of several plants such as *Litsea cubeba (Litsea)*, lemongrass, clove basil, and lemon. May Chang is used both as a food source and medicine in China. Citral has a rich lemon aroma and is usually used as a flavouring agent, preservative, and fragrance agent (Choi et al. 2009; Piorkowski and McClements 2014). It is highly valued for its anti-inflammatory (Bachiega and Sforcin 2011), antifungal (Silva et al. 2008), antitumor (Shi, Zhao, et al. 2016), and antibacterial activities (Somolinos et al. 2010; Shi, Song, et al. 2016, Shi, Zhao, 2016). It has been reported to possess carminative, diuretic, deodorizing, and central nervous system-stimulating effects (Carbajal et al. 1989).

Citral activates peroxisome proliferator-activated receptor (PPAR) α and PPARγ, suppresses cyclooxygenase-2 expression (Katsukawa et al. 2010), and inhibits lipopolysaccharide-induced acute lung injury by activating PPAR-γ (Shen et al. 2015). PPARα is a member of the nuclear receptor super-family of ligand-activated transcription factors, and it functions as an obligate heterodimer with retinoid X-receptor alpha. PPARα activation partially affects the expression and activity of cytochrome P450 (CYP450) enzymes (Shaban et al. 2005; Zhao et al. 2006). Moreover, citral induces hepatic peroxisomal and microsomal enzymes (Roffey et al. 1990; Li et al. 2017).

As a potential drug candidate, citral is prone to drug–drug interactions; however, data on its interactions with drugs are limited. Therefore, this study evaluates the effect of citral on the expression and activities of hepatic CYP450 enzymes in mice thereby reducing the risks associated with its clinical use.

## Materials and methods

### Materials

Phenacetin, dextromethorphan, chlorzoxazone, and testosterone were obtained from Shanghai Aladdin Biochemical Technology Co. Ltd., Shanghai, China. Citral (>99% pure) was purchased from Sigma-Aldrich Co. LLC. (St. Louis, MO). The liver microsomal incubation system was purchased from Wuhan Puleite Biomedical Technology Co. Ltd., Wuhan, China. The reagents for molecular biology were purchased from Bio-Rad Laboratories Inc., Hercules, CA. All analytical kits were purchased from Nanjing Jiancheng Biology Engineering Institute, Nanjing, China. All other chemicals and reagents were of analytical grade and obtained commercially.

### Animals

Fifty ICR male KM mice (6 weeks, 18–22 g) were purchased from Dossy (Chengdu Dossy Biological Technology Co. Ltd, Chengdu, China). The animals were housed in polypropylene animal cages according to different groups in a ventilated room maintained at 25 ± 2 °C and 70 ± 10% relative humidity, on a 12 h light/dark cycle. Water and food were provided *ad libitum*. All animal experiments were conducted in accordance with the principles of good laboratory animal care and performed in compliance with the Animal Ethics Review Committee of Chengdu Medical College.

The mice were acclimatized to the environment for 7 d before initiating the treatment. They were then divided into the following five groups (*n* = 10 each): negative control (group C: 0.9% saline), Tween-80 (group T: 0.5% in water), citral 20 mg/kg (group L), citral 200 mg/kg (group M), and citral 2000 mg/kg (group H). Citral was dissolved in water, followed by the addition of Tween-80 (0.5% v/v), and intragastrically administered to mice for 3 d. The mice were acclimatized to the environment for 7 d before initiating the treatment.

### Sample collection

On the third day of the experiment, following an overnight fast of 8 h, the mice in all the groups were anesthetized using ether. The blood samples were collected by cardiac puncture into non-heparinized tubes, and the serum was immediately separated for analysis.

Liver microsomes and cytosol fractions were prepared by differential centrifugation (Goossens et al. 2013). The liver was excised, rinsed with ice-cold saline (0.9% NaCl w/v), weighed, and homogenized in a 0.05 mM Tris/KCl buffer (pH 7.4). The homogenate was centrifuged at 10,000 *g* at 4 °C for 30 min, and the supernatant was centrifuged at 105,000 *g* at 4 °C for 60 min. The pellet was reconstituted with 0.05 mM Tris/KCl buffer (pH 7.4) and stored at −80 °C until analysis. The protein content in the liver microsomes was determined using the Bradford protein assay kit (Shanghai Beyyotime Biological Technology Co. Ltd., Shanghai,  China). The liver microsomes were used to analyze the activity of CYP450 enzymes.

A portion of the liver tissue (0.5 g) was snap-frozen in liquid nitrogen and stored at −80 °C until mRNA extraction and gene expression analysis.

### Biochemical analyses

Glutamic pyruvic transaminase (GPT) and glutamic oxaloacetic transaminase (GOT) activities were analyzed according to the manufacturer’s instructions.

### Antioxidant activity

The antioxidant activity of citral was evaluated by measuring the total antioxidant capacity (T-AOC), total superoxide dismutase (T-SOD) activity, malondialdehyde (MDA), glutathione (GSH), nitric oxide (NO), and hydroxyl radicals in the serum of mice according to the manufacturer’s instructions for the corresponding kits.

### Histology

The histopathological evaluation of the liver was performed by fixation of the liver tissue sections in a 10% neutral-buffered formalin solution for 1 week. The tissues were stained with haematoxylin and eosin for microscopic examination. All observations were made manually in a blinded manner using a light microscope with ×5, ×10, ×20, and ×40 objective lenses.

### RNA extraction and gene expression in the liver by real-time polymerase chain reaction

RNA was extracted and gene expression in the liver tissue was analyzed as previously described (Iovu et al. 2012). The quality and quantity of the extracted mRNA were determined using an ultraviolet (UV)-Vis spectrophotometer (NanoDrop 2000 UV–Vis spectrophotometer, Thermo Scientific, Waltham, MA). The expression of the following target genes was analyzed: CYP450 1A2, 2D22, 2E1, and 3A11. The housekeeping gene glyceraldehyde 3-phosphate dehydrogenase (GAPDH) was used for data normalization (Table 1). All primers used in this study were synthesized by the Beijing Genomics Institute (Beijing, China). An arbitrary scale was used for statistical comparisons. Melting curves and polymerase chain reaction (PCR) efficiency were used as the standard quality criteria for each real-time (RT)-PCR run.

**Table 1.** PCR primers used for gene expression analysis.

Target	Primer sequence (5′–3′)	Primer size (bp)
CYP450 1A2	CAGGAGCACTACCAAGACTTCATGGATCAACCTCTGCACGTT	115
CYP450 2D22	CAGTGTCCAGATGGCAGAAGGACAGGTTGGTGATGAGG	135
CYP450 2E1	TTCCCTAAGTATCCTCCGTGACGTAATCGAAGCGTTTGTTG	194
CYP450 3A11	ACAAACAAGCAGGGATGGACGGTAGAGGAGCACCAAGGTG	150
GAPDH	GATGGAAGGTCGGTGTGATGAAGGGGTCGTTGATGG	131

### Activities of CYP450 1A2, 2D22, 2E1, and 3A11 enzymes

CYP450 1A2, 2D22, 2E1, and 3A11 enzyme activities were assessed as reported previously, with slight modifications (Rao et al. 2003; Juřica et al. 2010). All microsomal incubations were carried out for 60 min at 37 °C in a final volume of 500 μL of pooled microsomes (1 mg/mL protein) and an NADPH-regenerating system. A mixture of magnesium chloride (MgCl_2_; 10 mM), glucose-6-phosphate (10 mM), NADP^+^ (1 mM), and pyruvate dehydrogenase (2 U/mL) was used for regenerating NADPH. Probe substrates specific for the four CYPs were added during microsomal incubations. The final concentration of the organic solvent (methanol or acetonitrile) used in the reaction was adjusted to 1% (v/v). All incubations were terminated by adding 500 μL ice-cold acetonitrile containing tinidazole (20 ng/mL) as internal standard. Then, the samples were thoroughly mixed and centrifuged (18,000 *g* at 4 °C for 10 min) to obtain supernatants, and 10 μL of the supernatant was subjected to cocktail high-performance liquid chromatography (HPLC) analysis. The relevant enzyme activities were evaluated based on the reduction in concentrations of the following four probe substrates: phenacetin, dextromethorphan, chlorzoxazole, and testosterone for CYP450 1A2, 2D22, 2E1, and 3A11, respectively. HPLC analyses of phenacetin, dextromethorphan, chlorzoxazole, testosterone, and tinidazole (internal standard) were performed on an Agilent 1260 series instrument (Agilent Technologies, Palo Alto, CA) with a diode array detector at 230 nm. An Agilent reversed-phase C18 column (ZORBAX SB-C18, 4.6 × 250 mm, 5 μm) with a C18 guard column was used with a mobile phase containing acetonitrile and water (0.01 M acetic acid) in the ratio of 40:60 at 30 °C. The flow rate was 1 mL/min.

### Data analysis

The incubation assays were carried out in triplicate. Statistical analysis of the data was performed by one-way analysis of variance using SPSS 19.0 (SPSS, Chicago, IL), and the least significant differences were calculated after comparing the mean values with those of the control group.

## Results

### Effects of citral on biochemical indices

**Figure 1.**
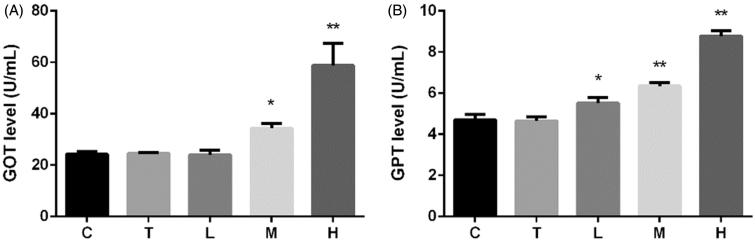
Effect of citral on serum (A) glutamic oxalacetic transaminase (GOT) and (B) glutamic pyruvic transaminase (GPT) levels. *p < 0.05; **p < 0.01, significantly increased versus control. C (control), T (Tween), L (low), M (middle), and H (high).

Citral significantly increased serum GOT (SGOT) and serum GPT (SGPT) levels (Figure 1). SGOT and SGPT levels in group H were 2.4 and 1.8 times higher than those in both groups C and T, respectively. These levels increased in group M; however, the SGOT level remained unaltered in group L.

### Effects of citral on antioxidant indices

**Figure 2.**
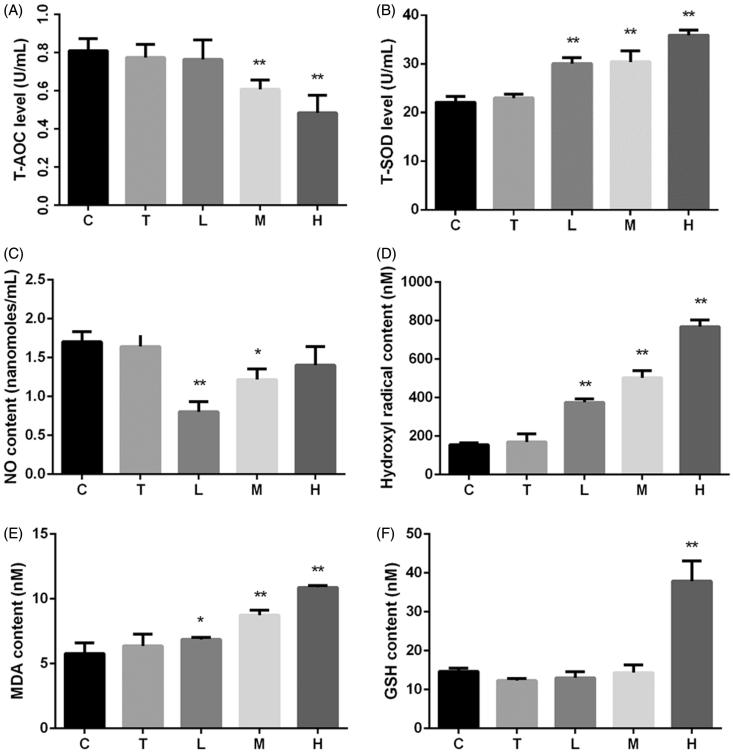
Effects of citral on (A) total antioxidant capacity (T-AOC), (B) total superoxide dismutase (T-SOD), (C) nitric oxide (NO), (D) hydroxyl radicals, (E) malondialdehyde (MDA), and (F) glutathione (GSH). *p < 0.05; **p < 0.01, significantly increased versus control. C (control), T (Tween), L (low), M (middle), and H (high).

Citral-induced oxidative stress levels in mice are shown in Figure 2. Although T-SOD level increased at all three doses of citral, the T-AOC, which is related to the levels of MDA and hydroxyl radicals, decreased significantly in groups M and H. NO content decreased significantly in groups L and M; however, the decrease inversely correlated with the dose.

### Effects of citral on histology of the liver

**Figure 3.**
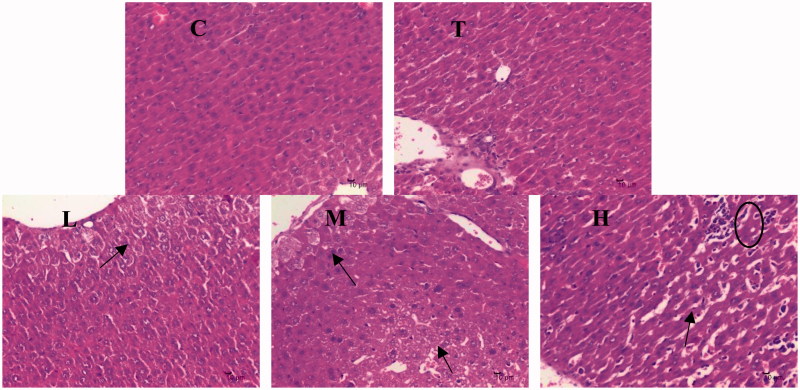
Effects of citral on histopathology. C (control), T (Tween), L (low), M (middle), and H (high).

Citral-induced changes in histology of the liver are shown in Figure 3. Hepatocytes were devoid of obvious lobulations and the arrangement of hepatic cord was disordered in the M and H groups. Occasionally, a small amount of vacuolar degeneration of central venous hepatocytes was observed in the L, M, and H groups.

### Effect of citral on the mRNA expression of CYP450 enzymes

**Figure 4.**
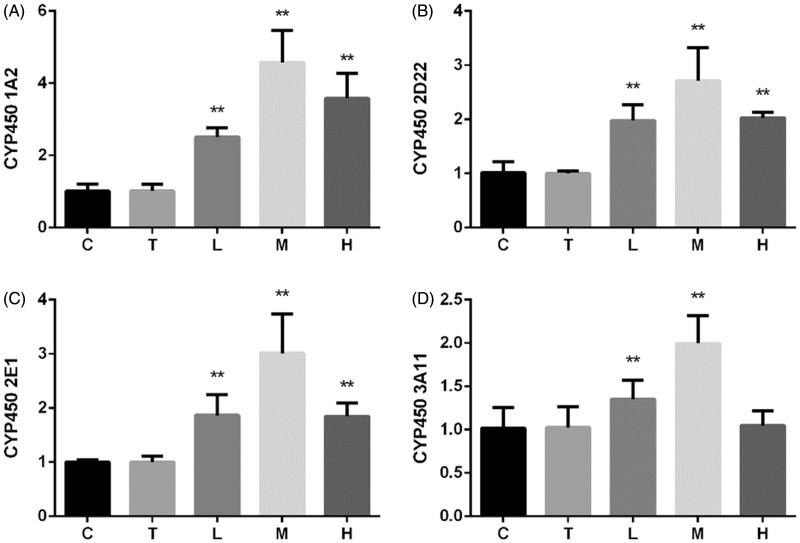
Relative mRNA expression of CYP450 (A) 1A2, (B) 2D22, (C) 2E1, and (D) 3A11 enzymes. *p < 0.05; **p < 0.01, significantly increased versus control. C (control), T (Tween), L (low), M (middle), and H (high).

The mRNA expression levels of CYP450s are shown in Figure 4. Citral significantly increased the mRNA expression levels of CYP450 1A2, 2D22, and 2E1 at all the three doses. The increase was the greatest in group M. CYP450 3A11 expression increased in groups L and M, while no change was observed in group H.

### Effect of citral on activities of CYP450 enzymes

**Figure 5.**
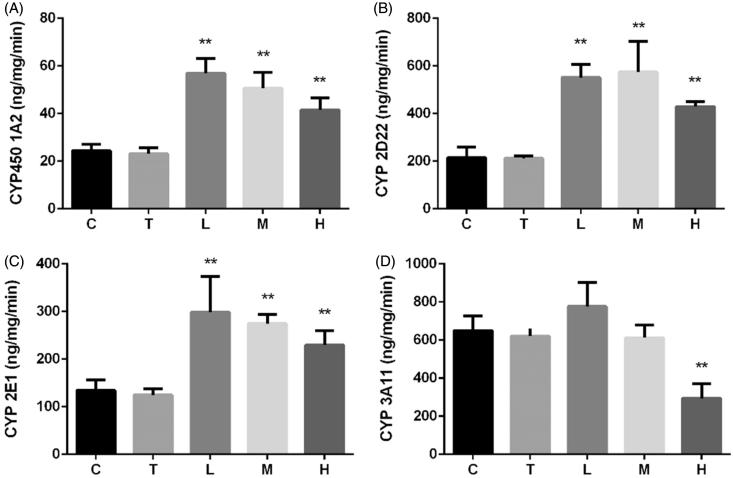
Effect of citral on activities of CYP450 (A) 1A2, (B) (C) 2D22, 2E1, and (D) 3A11 enzymes. *p < 0.05; **p < 0.01, significantly increased versus control. C (control), T (Tween), L (low), M (middle), and H (high).

Citral-induced changes in activities of CYP450s were similar to changes in their mRNA expression (Figure 5). Although citral induced CYP450 1A2, 2D22, and 2E1 activities, the increase inversely correlated with the dose. CYP450 3A11 activity significantly decreased in group H.

## Discussion

There has been a growing interest in investigating the potential of natural products as drugs for the treatment of various diseases. However, these drug candidates need to be evaluated for their toxicity. In this study, we measured the levels of two liver-specific enzymes, SGOT and SGPT, which are most commonly used as markers of hepatotoxicity (Freitag et al. 2015). Therefore, the analysis of these biomarkers is important for the identification of drug-induced liver lesions (Kumar et al. 2010; Ortega-Alonso et al. 2016). The essential oil of lemongrass (100 mg/kg) does not affect the relative liver weight in mice (Costa et al. 2011). Nevertheless, citral (2.4 g/kg) is reported to significantly increase the absolute and relative liver weights in rats (Jackson et al. 1987). Our results shown in Figures 1 and 2 reveal that citral causes considerable liver injury. Citral at a dose of 2000 mg/kg increased SGOT and SGPT levels to 2.4 and 1.8 times, respectively, that of the control group. Although citral is reported to have a protective effect on acetaminophen-induced liver toxicity at doses of 125–500 mg/kg (Uchida et al. 2017), we suggest that higher doses may have a toxic effect on the liver and should be avoided.

A balance exists between oxidation and antioxidation processes in the body, and any disturbance in this balance affects the normal physiological functions (Huang et al. 2005). Many natural substances, such as vitamin C, vitamin E, selenium, and carotenoids, exhibit antioxidant activities (Monsen 2000; Johnson et al. 2003). Citral is reported to possess good antioxidant activity *in vitro* (Jiang 2012; Bouzenna et al. 2017). In our *in vivo* study, the serum antioxidant indices indicated that citral increased T-SOD and GSH levels; however, T-AOC decreased significantly at high doses of citral. Moreover, the hydroxyl radical and MDA contents significantly increased at all three doses of citral. Therefore, we hypothesize that citral is a weak antioxidant or has low antioxidant capacity *in vivo*. High-dose citral induced oxidative stress in mice in this study.

CYP450 are key enzymes involved in drug–drug interactions, and their expression changes in various physiological and pathological states (Renton 2001). Inflammation and infection are the main factors affecting the expression of liver enzymes (Renton 2004; Aitken et al. 2008) besides other factors such as sex, age, and hormonal and diurnal rhythms. CYP450 1A2, 2D22, 2E1, and 3A11 account for more than 50% of all drug-metabolizing enzymes in the liver and metabolize more than 50% of all the drugs used clinically (Pan et al. 2014). Oxidative stress and NO are two important factors regulating the expression of CYP450 enzymes. An increase in oxidative stress and NO suppresses CYP450 expression (Barouki and Morel 2001; Vuppugalla and Mehvar 2004). Our results shown in Figure 3 indicate that citral induces the mRNA expression of several CYP450 enzymes, and the greatest effect is observed at a dose of 200 mg/kg. A dose of 2000 mg/kg had a lower induction effect on the mRNA expression of CYP450 1A2, 2D22, and 2E1 than a dose of 200 mg/kg. In addition, citral had no effect on the mRNA expression of CYP450 3A11 at a dose of 2000 mg/kg. The changes in CYP450 expression correlated with the antioxidant effects shown in Figure 2, particularly with the effect on NO levels. Citral at 200 mg/kg induced the mRNA expression of CYP450 enzymes to a greater extent than at 2000 mg/kg, probably because significant liver injury was induced at 2000 mg/kg.

The cocktail approach is most commonly used for assessing *in vitro* activity (Bosilkovska et al. 2014; Spaggiari et al. 2014). The changes in concentrations of different CYP450 probe substrates are measured to determine the corresponding enzyme activities (Sun et al. 2014). Previous studies have reported that citral significantly increased total CYP450 levels in mice and rats (Jackson et al. 1987; Roffey et al. 1990). Our results shown in Figure 4 are consistent with the findings of previous reports. Because high-dose citral induced considerable liver injury and oxidative stress, the increase in the activity of several CYP450s inversely correlated with the dose. Citral at a dose of 2000 mg/kg induced the activities of CYP450 1A2, 2D22, and 2E1, whereas CYP450 3A11 activity was suppressed.

An assessment of CYP450 enzyme activity is essential to characterize phase I metabolism in biological systems or to evaluate the inhibition/induction properties of xenobiotics. The major CYP450 isoforms such as 1A2, 2C9, 2C19, 2D6, and 3A have been primarily studied (Zhou et al. 2009; Samer et al. 2013). CYP450 isoenzymes are responsible for the metabolism of approximately 90% of drugs (Genovese et al. 2011). CYP450 3 A is reported to be the most abundant CYP450 subfamily in the human liver. It represents 50–70% of the total CYP450 enzymes and is responsible for the metabolism of more than 50% of prescribed pharmaceuticals (Urquhart et al. 2007). Low-dose citral induced the expression of most of the CYP450 enzymes, while high-dose citral significantly decreased CYP450 3A11 expression. Therefore, the dose of citral is very important. Low-dose citral will decrease the efficacy of concomitantly administered drugs, whereas high-dose citral will lead to drug accumulation and poisoning. And more research is needed to clarify the mechanism.

## Conclusions

In summary, the present study showed that treatment with citral induces significant liver injury at a dose of 2000 mg/kg. Besides inducing oxidative stress, citral improved the activity of cellular antioxidant enzymes. Citral caused liver injury at high doses and induced the mRNA expression and activities of CYP450 1A2, 2D22, 2E1, and 3A11 in mouse liver at low doses. Thus, the dose of citral must be carefully selected. High-dose citral significantly decreases the activity of CYP450 3A11. As CYP450 enzymes are the most important enzymes responsible for the biotransformation of numerous drugs in humans, further studies are needed to investigate possible drug-drug interactions if citral is to be used as a drug or food additive.
